# Measurement system with real time data converter for conversion of *I*^2^*S* data stream to UDP protocol data

**DOI:** 10.1016/j.heliyon.2020.e03760

**Published:** 2020-04-09

**Authors:** Zoltan Vizvari, Attila Toth, Zoltan Sari, Mihaly Klincsik, Bojan Kuljic, Tibor Szakall, Akos Odry, Kalman Mathe, Imre Szabo, Zoltan Karadi, Peter Odry

**Affiliations:** a Department of Environmental Engineering, Faculty of Engineering and Information Technology, University of Pécs, Boszorkány str. 2, H-7624 Pécs, Hungary; b Institute of Physiology, Medical School, University of Pécs, Szigeti str 12, H-7624 Pécs, Hungary; c Department of Information Technology, Faculty of Engineering and Information Technology, University of Pécs, Boszorkány str. 2, H-7624 Pécs, Hungary; d Department of Mathematics, Faculty of Engineering and Information Technology, University of Pécs, Boszorkány str. 2, H-7624 Pécs, Hungary; e College of Applied Sciences, Subotica Tech, Marka Oreškoviċa 16, 24000 Subotica, Serbia; f Institute of Information Technology, University of Dunaujvaros, Táncsics M. str. 1/A, H-2401 Dunaújváros, Hungary; g Faculty of Engineering and Information Technology, University of Pécs, Boszorkány str. 2, H-7624 Pécs, Hungary; h Department of Behavioural Sciences, Medical School, University of Pécs, Szigeti str 12, H-7624 Pécs, Hungary

**Keywords:** Electrical engineering, Neuroscience, Electrical system, Electrical system planning, Systems neuroscience, I2S protocol, TCP/IP protocol, Protocol converter, Scalable system

## Abstract

A central goal of systems neuroscience is to simultaneously measure the activities of all achievable neurons in the brain at millisecond resolution in freely moving animals. This paper describes a protocol converter which is part of a measurement acquisition system for multichannel real time recording of brain signals. In practice, in such techniques, a primary consideration of reliability leads to great necessity towards increasing the sampling rate of these signals while simultaneously increasing the resolution of A/D conversion to 24 bits or even to the unprecedented 32 bits per sample. In fact, this was the guiding principle for our team in the present study. By increasing the temporal and amplitude resolution, it is supposed that we get enabled to discover or recognize and identify new signal components which have previously been masked at a “low” temporal and amplitude resolution, and these new signal components, in the future, are likely to contribute to a deeper understanding of the workings of the brain.

## 1 Introduction

Since the nervous systems show highly complex dynamics, one of the greatest scientific challenges is the monitoring the electrical activities of neurons within functioning brains [1], [2], [3], [4], because the nervous systems show highly complex dynamics. This complexity originates from single-neuron spiking activity (SA) to various frequency bands in broadband local field potentials (LFPs) that have been defined mostly by their frequency bands. However, even the operation in which electrodes are implanted into the appropriate part of the brain of an experimental animal already interferes with the “naturalness of life”, and simultaneously, the content of these signals, representing a response to particular stimuli in which a given experimental animal is exposed to, is also modified by interference.

In practice, many various systems regarding transmitting the measured brain signals have been created. These systems for transmission and processing can be divided into two greater groups:

- The first group consists of systems where only measurements are done upon the head of an animal (with or without digitalizing the signal, but without processing it) and data are transmitted real time to the stationary unit [5], [6].
- The second group consists of systems, of which, in addition to performing measurements upon the head of an animal, locally extract useful information (they extract, for instance, information in reference to spikes, or do compression with a loss) which is sent to the stationary unit [7], [8]. A sub-group of these systems represents those solutions where data archiving is performed on an SD card and is mounted onto the body of the animal, and reading the data is performed while charging the battery [9].

The equipment and the supplementary software packets used to extract brain signal spikes in neurobiological research rely on relatively low sampling rates per channel, being of the order 1-10 ksample/s and a signal resolution of 10-16 bits [6], [9], [10], [11], [12]; therefore, software packages in support of processing these types of signals have also been created mainly regarding this range of data rates. In [13], [14], there is a detailed discussion of the required sampling rates for various types of brain activity signals, based on the sampling theorem. An overview of sampling rates and resolutions for particular systems is given in [11], [15].

In the present research, in the primary signal processing upon the head of the animal, cutting-edge technology has been used for digitalizing the audio signal and components as well as for transmission to the stationary unit [16]. Similarly, audio processors have been used in the stationary unit regarding pre-processing [16]. Compared to analog transmission, digital transmission has shown better results both in the transmission quality and in the dimensions, weight, energy consumption and autonomy (higher resistance to radio noises) of the system for the same number of measured channels.

## 2 The implementational principle of the protocol converter

**Figure 1:**
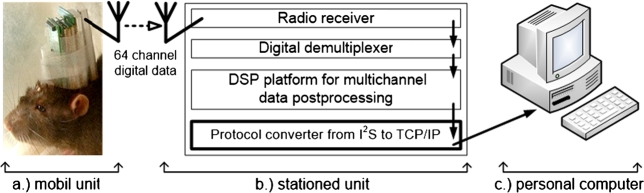
Block diagram of the acquisition system.

The realized system for the acquisition and archiving of brain cell signals has been developed on the following principle: the brain cell signals were locally digitalized [5] upon the head of the animal (Fig. 1.a); the multiplexed signal was wirelessly transmitted to the stationary unit of the system (Fig. 1.b) and, after processing and conversion, sent the resulting data for archiving to a personal computer (Fig. 1.c). The structure of the system is depicted in the block diagram in Fig. 1, while the bolded box represents the part of the system that the present paper deals with, i.e., the protocol converter from $I^{2}S$ to TCP/IP.

The full system consists of three units:

- The first part is the mobile unit of the system (Fig. 1.a). The sources of the signal are the brain cells of the experimental animal: mouse, rat, monkey, etc. The probes for collecting the data are of invasive type [5]. The measured signals are of low intensity, whose A/D conversion is done locally, upon the head of the animal, to weaken the annoying effect of noise [17]. The number of measuring channels in the final system which is multiplexed, is 64, the sampling frequency per channel is 24000 Hz, and the sampling resolution is 24 bits. The digitalized data at the place of measurement following A/D conversion are represented in a multichannel $I^{2}S$ format (audio converters usually use this protocol). The data are multiplexed in an FPGA circuit and are sent wirelessly to the stationary unit of the system [18], [19]. Details of the acquisition system such as the cross-point switch arrays [20], low-noise power supply [17] and the test generator of the mobile unit of the system [21] are to be found in the cited literature. When working with the brain signals and other biomedical measurements with multichannel instruments, a special care must be invested in the galvanic isolation and low noise power supply [22].
- The stationary unit of the device (Fig. 1.b) is used for receiving, de-multiplexing and reconstructing the individual $I^{2}S$ signal components. The Multiple Instruction Multiple Data (MIMD) multi-processor [23], digital signal processing (DSP) unit using powerful TAS3108 audio processors [24] can also be found in the stationary unit [16]. The DSP block consists of 36 TAS3108 audio processors used for pre-processing the signal in real time, i.e., filtering the incoming data. The group of the 36 audio processors quite adequately demonstrates flexibility, notably, regarding its connectivity, and the structure of a single processor highlighting their mutual connection, which is software-controlled. The TAS3108 audio processor itself has a particularly flexible structure regarding the simultaneous digital processing of eight mutually independent input channels. During processing, there is a possibility of mixed processing regarding the multiple signals per channel. The DSP blocks have been designed in such a way that, depending upon the type and field of analysis, the characteristics of filtering can be software-defined prior to initiating the measurements individually for each of the 64 channels. The data on the output of the DSP blocks are in the $I^{2}S$ format. This technology makes it possible to take broadband neural recordings at a 24-bit resolution, and when adding an additional 8 bits, other important information (specific behavioral responses, extension of spatial and temporal information) can be attached without further data loss.
  The last sub-functional block of the stationary unit of the system (Fig. 1.b, bolded box) is the protocol converter whose task is to convert, in real time, the synchronous data stream of the multichannel $I^{2}S$ signal into asynchronous UDP packets. The present paper describes an essential solution associated with the problem of implementing such a protocol converter. Single Instruction Multiple Data stream (SIMD) processor can also accomplish MIMD operations that approach tend to take a lot more time. SIMD processors are simpler, smaller and faster than MIMD, but they must perform complex operations sequentially, while MIMD processors perform complex operations concurrently [25].
- The third unit of the system is the personal computer (Fig. 1.c), which is used for archiving and visualizing the data. A dedicated computer network has been used for connecting the computer with the measuring system. At the provided measuring parameters, 50 hours of experimental data can be stored on the 1TB hard disk memory.

**Figure 2:**
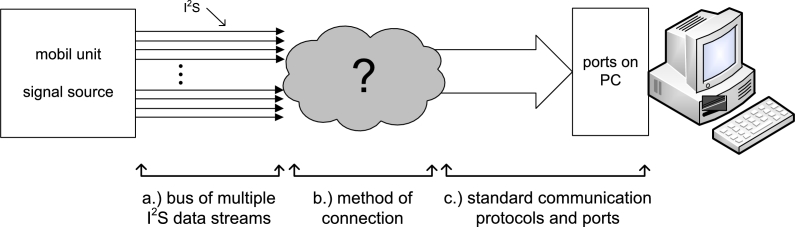
The problem solved is the connection of communication inside the measuring system.

The mobile measuring unit upon the head of the experimental animal (Fig. 1.a) is the source of the data stream and is organized in an $I^{2}S$ bus and is depicted in Fig. 2.a. These data need to be sent to the personal computer with the help of a standard computer protocol (Fig. 2.c). In regards to connectivity (Fig. 2.b) of the above-mentioned parts, a protocol converter has been created, described in the remainder of this paper, which represents an essential solution.

### 2.1 The $I^{2}S$ protocol

In the system, A/D converters and audio processors transmit data with the help of a often referred to, “Inter-IC-Sound” or shorter $I^{2}S$ synchronous serial protocol [26]. The physical level of the $I^{2}S$ protocol consists of three signals:

- SCK – (Serial Clock) continuous serial synchronous clock,
- SD – (Serial Data) digital audio content,
- WS – (Word Select) frame signalization.

Data transmission is synchronized with the SCK clock. The frequency ($f_{SCK}$) of this signal depends on the sampling frequency ($f_{s}$) of the audio data and the number of bits which needs to be transmitted in the WS frame.

(1) $$f_{SCK}=WS_{framelenght}\cdot f_{S}$$

In the $I^{2}S$ protocol, the SD signal is reserved exclusively for transmitting the digital informational content without compression. The data is transmitted in frames whose borders and middle point are marked by the WS signal. The SD line can be configured for transmitting 2, 4, 6 or 8 audio channels per frame. The data of the individual channels are placed in the frame one after the other using the TDM (Time Division Multiplex) method. Configurations for the serial transmission of multiple audio channels are marked as TDM2 (for 2 channels), TDM4 (for 4 channels), TDM6 (for 6 channels), and TDM8 (for 8 channels).

**Figure 3:**
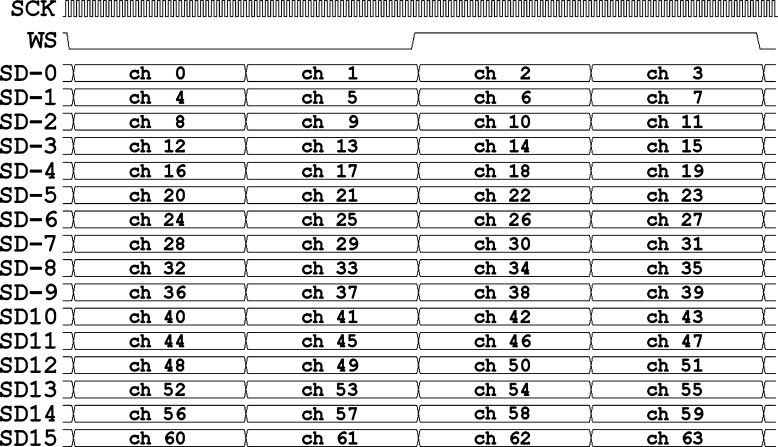
Time-proportional representation of the *I*^2^*S* signal of the bus in the device, with a distribution of data of the measuring channels.

In this case, the mobile unit of the device sampled the signals and wirelessly sent the values in a 24-bit format. In the stationary unit, each sample was extended into a 32-bit format during DSP processing. Data from the individual processors were merged in the bus, which contained a WS and an SCK line of each and 16 synchronized, parallel SD lines marked as SD0 to SD15. Each of those SD lines was in the TDM4 mode and transmitted the sampled data of 4 channels each (Fig. 3.), so the total number of channels was 64.

In the mobile unit of the system, the employed high-quality audio A/D converters provide the measurement data via $I^{2}S$ standard. The data preprocessing is performed with digital signal processors (DSPs) which support left-aligned, 32 bit, TDM4 $I^{2}S$ data streams. Our coding system was adapted to the mobile unit, which forwards the data of 64 channels in 16 parallel $I^{2}S$ data streams. The data which characterize the bus of the $I^{2}S$ device are as follows (The following settings were applied because we were looking for simple, easy-to-implement solutions. Our additional goal is to collect data with the smallest possible pulse signal shape distortion, which allows us to record spikes that have not been detected by the development of subsequent new processings.):

1. number of bits in the data: *data length* = 32 *bits*,
2. sampling frequency: $f_{s}=24000\;\text{Hz}$,
3. number of measuring channels: $ch_{NO}=64$ channels,
4. number of measuring channels transmitted in one $I^{2}S$ SD line:
  $TDM_{NO}=4$,
5. a multiple $I^{2}S$ stream contains $SD_{NO}=ch_{NO}/TDM_{NO}=64/4=16$ synchronous, parallel SD lines and only one SCK and one WS line,
6. data sampling and transmission have been synchronized, i.e., the period of the WS signal is the same as the sampling period: TWS=1/fs=1/24000Hz,
7. in each WS frame, 4 words of data are transmitted (Fig. 2.):
  (2) $$WS_{framelenght}=TDM_{NO}\cdot data\;length=4\cdot 32\;\text{bits}=128\;\text{bits}$$
8. in support of the synchronous transmission, there must be a clock representative of the SCK signal for each bit of data within the frame; in accordance with Eq. (1), the frequency of SCK is:
  (3) $$f_{SCK}=WS_{framelenght}\cdot f_{s}=128\cdot 24000=3072000\;\text{Hz}$$
  and the period of the SCK line is:
  (4) $$T_{sck}=\frac{1}{f_{SCK}}\approx 326\;\text{ns}$$
9. the bus consists of $SD_{NO}=16$ parallel lines of data, i.e., the quantity of data in one WS block is:
  (5) $$data\;in\;one\;WS=SD_{NO}\cdot WS_{framelenght}=16\cdot 128\;\text{bits}=256\;\text{bytes}$$
10. minimum bandwidth of the communication channel, necessary to transmit the data stream of the $I^{2}S$ bus:
  (6) $$stream\;data\;volume=SD_{NO}\cdot f_{SCK}=16\cdot 3072000=49.152\;\text{Mbit}/\text{s}$$

The data stream of the mobile unit (49.152 Mbit/s Eq. (6)) is controlled with a traffic shaping token-buket algorithm. This is a monotonic, edge-controlled task, which does not require the interpretation of data passing through this phase. Therefore, this part of the protocol converter was realized in speed efficient fashion with electronic components (complex programmable logic device (CPLD) and FIFO RAM). The protocol converter transmits the content of $I^{2}S$ data stream to the archiving PC with UDP packets. Similarly, this task doesn't require the interpretation of data, but both the change of data format and implementation of TCP/IP stack are realized with an embedded system as a cost effective solution. In this case, the protocol converter needs to read the data of the $I^{2}S$ bus, to convert the protocol of the measuring device into a UDP protocol and send the UDP packet to the computer.

### 2.2 A model of the protocol converter

**Figure 4:**
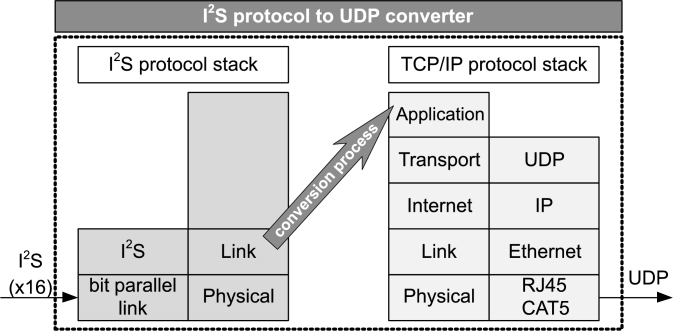
A model of data conversion on a dual OSI protocol stack.

In Fig. 4, two OSI protocol stack models are shown: the $I^{2}S$ stack of the incoming stream (left part) and the TCP/IP stack of the outgoing data (right part). The two stacks are connected by the data conversion process, which contains the transformation of the data and frame representations within the $I^{2}S$ bus into the data frames of the UDP packet.

**Figure 5:**
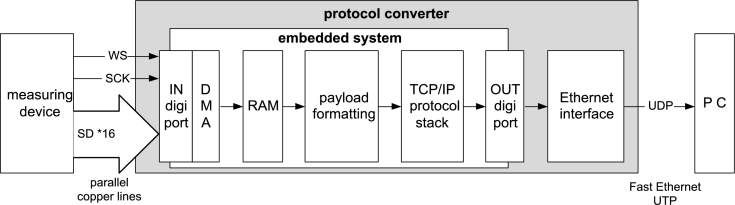
Block diagram of the protocol converter, with the controlling block implemented as an embedded system.

After the model to be used for solving the problem was created, the functional model of the protocol converter was adjusted to the problem at hand and was further defined. In this way, the block diagram of the planned protocol converter has been effectively created (Fig. 5.).

Fig. 5 depicts the structure of embedded Protocol Converter, which is also highlighted by the thick frame in the bottom of Stationary unit depicted in Fig. 1b. The data collecting system supplies 16 data threads to the input, where each thread consist of 4 channel $I^{2}S$ data streams. As a result, the 64 independent measurement channels can be processed. The data are received in the control unit of embedded protocol converter (white rectangle in the middle of the figure) via digital input ports (IN digital ports). The data on I/O ports access the dual access RAM of the embedded system via DMA (Direct Memory Access) controller.

Standard UDP packets are formed from the data received in the RAM with a Payload Formatting processor-process. The protocol converted can be realized both in one and multi channel format. By increasing the number of processing units of the protocol converter, the processing system provides wider time slots for protocol conversation: in case of one protocol converter 208.3μs, while in case of two protocol converter 458.3μs time slots are allowed. The UDP packet contains a user header beside the mandatory fields, which is used for synchronization and packet following.

The UDP packets are handled with a software-based TCP/IP stack. The output processor-process transmits the UDP packets to the Ethernet PHY unit via the digital output ports of the embedded system (OUT digital ports). This unit – right side of the gray rectangle – transmits the UDP data packets to the PC via twisted pair UTP transmission medium (FastEthernet standard).

The cumulative data stream speed is determined by the sampling frequency of each channel of the measurement system, which is 48 Mbit/s data stream in our case. Based on Eq. (11), 4800 UDP packet is generated in each second from the data stream. For the creation/transmission of one UDP data packet less than Eq. (10) or Eq. (19) time slots are available. The creation of one UDP packet includes reception from DMA unit, UDP conversion and Ethernet data transmission. If the packet generation is slower than data reception speed, then data congestion would result in packet loss, independently of the available buffer size.

The time limit could vary (increase of decrease) if different channel number, sampling frequency, bit length or protocol converter control unit is employed.

The maximum length of the UDP packet is limited by a standard. The total amount of payload, data with the header of the application, can reach a maximum 1472 bytes. Using the values in Eq. (5), it can be calculated how many complete WS frames of the bus can fit into a UDP packet of maximum length:

(7) $$UDP_{payload}=\frac{1472\;\text{bytes}}{data\;in\;one\;WS\;block}=\frac{1472\;\text{bytes}}{256\;\text{bytes}}\approx$$

(8) ≈5(WSblockofthebus)=1280bytes

which means the maximum length of the UDP packet which can be formed from source data is 1280 bytes.

The time required for all data for forming a UDP packet to arrive:

(9) $$T_{UDP}=\frac{UDP_{payload}}{stream\;data\;volume}=\frac{1280\;\text{bytes}}{49.152\;\text{Mbit}/\text{s}}\approx 208.3\;\mu \text{s}$$

The frequency of forming and sending a UDP packet is:

(10) $$f_{UDP}=\frac{1}{T_{UDP}}=4800\;\text{Hz}$$

Apart from the usable data, the protocol converter also adds the header of the application to the UDP frame, the purpose of which is to, unequivocally identify the frame.

### 2.3 Platform for implementing the protocol converter

In the protocol converter, a small number of actions needs to be cyclically repeated, and for this task, a special purpose computer built into the device it controls is the most appropriate to use. The process of conversion must be performed in real time, i.e., the protocol converter has time limits within which it must complete the task of conversion. Based on the facts, it has been concluded in which an in-built system working in real time is the most effective way to control the protocol converter. For implementation, a MOD5270 [27] processor board with a ColdFire 5271 [28] 32-bit processor has been chosen. The module works in real time under the *μ*C/OS operating system, contains a library for working with the TCP/IP stack, has I/O ports, programmable DMA channels and a FastEthernet port.

### 2.4 Methods of page flipping and the token-bucket

Data integrity requires two separate processes: writing the incoming $I^{2}S$ data into the memory and reading the data from the memory with the aim of forming UDP packets. These processes access the data in the memory by using the efficient method of page flipping (sometimes also called ping-pong buffering). With this method, both processes use one memory buffer each. Upon finishing the work with their own current buffer, instead of copying the data, the processes of reading and writing mutually swap the buffer pointer values.

The processor can perform reading the data from the peripherals using the following procedures: polling, interrupt or DMA transfer. For the realized research, the DMA technique of transferring data blocks from the peripherals into the memory of the processor has been chosen.

For an efficient use of DMA transfer, instead of more frequent, individual data, it is desirable to have less frequent, larger blocks of data arriving to the input of the processor board. In accordance with Eq. (7), data needs to be collected into five WS blocks in support of forming the payload for one UDP packet. By using the token-bucket algorithm, it is possible to take a certain number of smaller incoming packets and form one larger packet from them, which is then forwarded onward. This algorithm does not change the order of the packets. For running a hardware token-bucket algorithm, the FIFO RAM chip SN74V293 [29] has been used.

**Figure 6:**
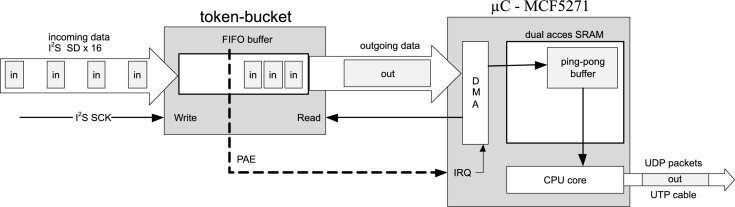
Hardware token-bucket with a ping-pong buffer.

The resulting token-bucket (Fig. 6.) corresponds to the FIFO buffer or queue structure. Small quantities of data which arrive to the input of the token-bucket are written into the buffer. When the written amount of data exceeds the limit (PAE in Fig. 6.), which signifies the amount of data required for forming a UDP packet is at its disposal, the hardware token-bucket triggers an interruption on the processor board. The triggered interruption routine will launch the DMA reading of data from the FIFO buffer and data transmission is performed into the internal ping-pong buffer.

## 3 Implementing the system

Implementation of the hardware was followed by performing measurements at the laboratory. During the measurements, a signal injector [21] which simulated the signals generated in the observed brain cells of the experimental animal and an oscilloscope for analyzing data transmission were used. The measurements have shown how the processor module of the chosen platform can perform in real time either DMA block data transfer at a rate of 49.152 Mbit/s (Eq. (6).) or forming and sending UDP data packets at the same rate. However, the processor module does not have enough processing time to perform both tasks.

**Figure 7:**
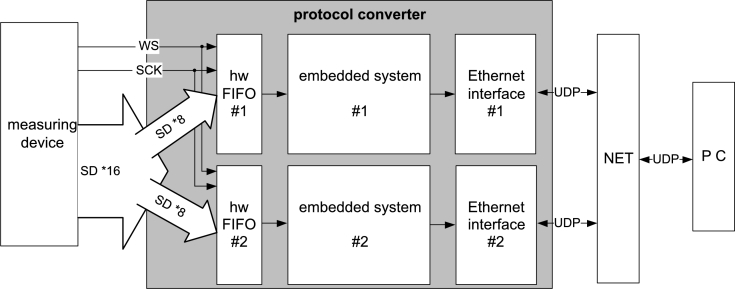
Block diagram of the protocol converter after scaling and re-design.

During the design of the measurement system, it was taken into account of which, in the future, a need will arise for transmitting data with even more channels and/or at a higher sampling rate. This is the reason why the protocol converter has also been designed to be scalable with minimal modifications. The solution with the token-bucket, DMA transfer, and the ping-pong buffer has proven itself, and as a result, the only bottleneck is the processing power. This is why a partial hardware and software re-design of the protocol converter has been performed, which increased its performance and functionality (Fig. 7.).

After scaling the protocol converter by a factor of 2, the following changes have taken place:

- The number of processor modules has been increased to two. The operation of the modules has been synchronized.
- The data of the $I^{2}S$ bus of 16 SD lines have been divided into two half buses of 8 SD lines, one for each module.
- Each of the new buses has its own hardware token-bucket.
- In the new architecture, the amount of data formerly put onto a load on one module since has been halved.
- An unequivocal marking of individual UDP packet pairs has been introduced, thus, sampled data can remain synchronized and archived on the computer at the same time as in the form of connected data.

Due to the re-design, the values of several parameters of the system underwent recalculation. Note: the notation of the changed parameters has received the extension ‘rd’ (re-design). The re-designed bus consists of two half buses with $SD_{rdNO}=8$ parallel data lines. The amount of data in one WS block is:

(11) $$data\;in\;one\;WS\;block_{rd}=SD_{rd_{NO}}\cdot WS_{framelenght}=$$

(12) =8⋅128bits=1024bits=128bytes

The minimum bandwidth of the communication channel required for the transmission of the data stream of the $I^{2}S$ half bus is:

(13) $$stream\;data\;volume_{rd}=SD_{rd_{NO}}\cdot f_{SCK}=8\cdot 3072000=24.576\;\text{Mbit}/\text{s}$$

Using the values from Eq. (11), it is possible to calculate how many complete WS frames of the bus can fit into a UDP packet after halving the amount of data on the bus:

(14) $$UDP_{rd_{payload}}=\frac{1472\;bytes}{data\;in\;one\;WS\;block_{rd}}=\frac{1472\;\text{bytes}}{128\;\text{bytes}}\approx$$

(15) ≈11(WSblockofthebus)=1408bytes

The time during which enough data arrive to the hardware FIFO to form a UDP packet:

(16) $$T_{rd_{UDP}}=\frac{UDP_{rd_{payload}}}{stream\;data\;volume_{rd}}=\frac{1408\;\text{bytes}}{49.152\;\text{Mbit}/\text{s}}\approx 458.3\;\mu \text{s}$$

The frequency of interrupt requests for DMA reading, conversion, forming and sending a UDP packet is:

(17) $$f_{rd_{UDP}}=\frac{1}{T_{rd_{UDP}}}=2182\;\text{Hz}$$

In addition to usable data, the protocol converter also adds the header of the application to the UDP frame, which serves to unequivocally identify the frame pairs of the separated bus. The header also contains the serial number of sampling which is used on the personal computer as a time stamp.

**Figure 8:**
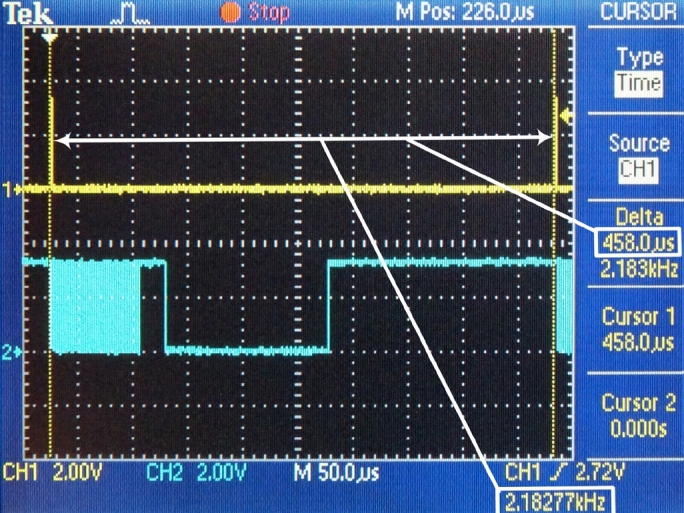
Frequency of interrupt requests for DMA transfer.

**Figure 9:**
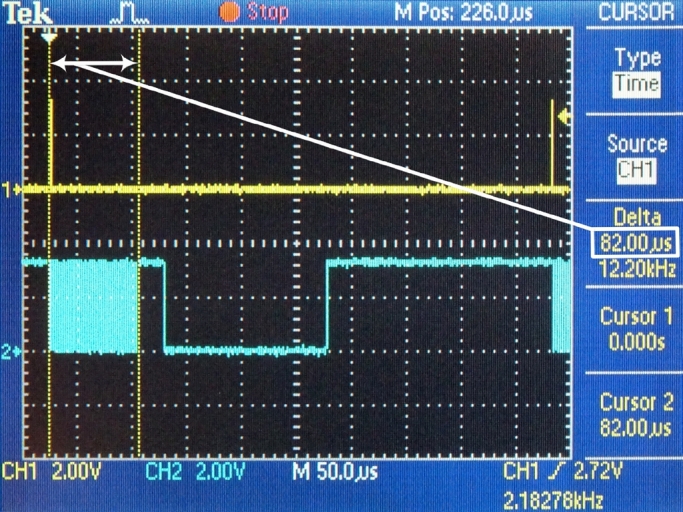
DMA transfer 1408 bytes.

**Figure 10:**
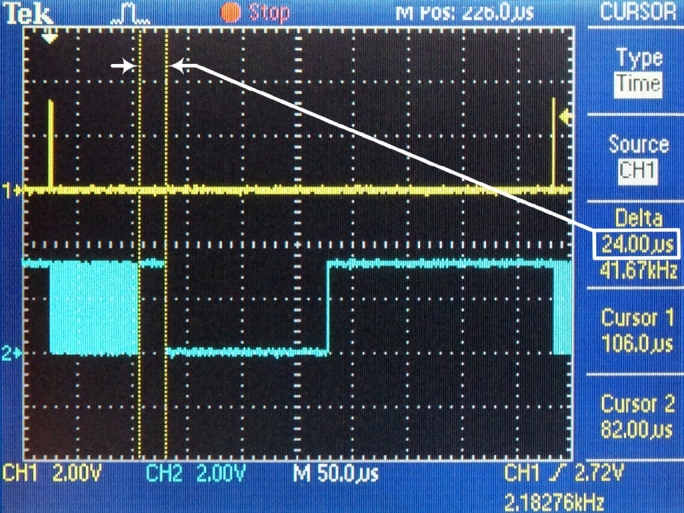
Forming a UDP packet and Ethernet frame.

**Figure 11:**
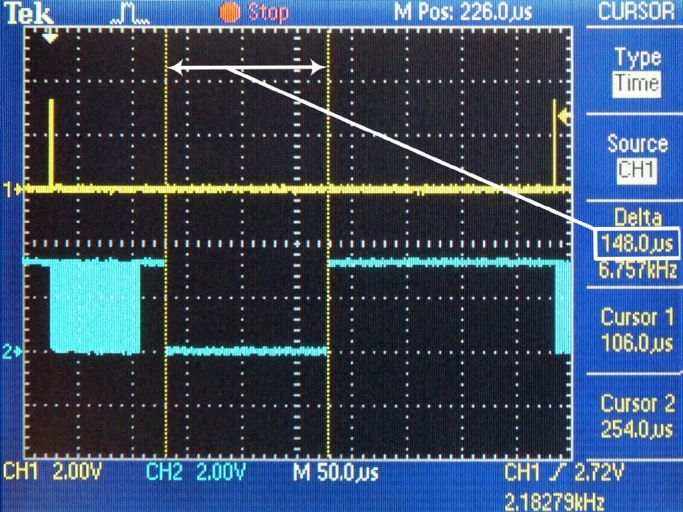
Transmission of the Ethernet frame.

The scaled protocol converter has been tested under laboratory conditions with the help of a dedicated signal generator [21] that simulated an experimental animal. Apart from reliability, the most important parameters are the time requirements of the individual tasks while receiving the data from the FIFO until sending the UDP packet via Ethernet. The time reserve of the implemented embedded system is also important information. During measurements, these parameters were read from the screen of the oscilloscope. Screenshots of the oscilloscope with commentaries are shown below (Fig. 8, Fig. 9, Fig. 10, and Fig. 11).

According to Eq. (17), the measurement system generates the certain amount of the measurement data, which can be used for forming a UDP packet with the frequency: $f_{rd_{UDP}}=2182$ Hz. This is also the frequency of interruption used to request a DMA transfer from the hardware FIFO into the internal ping-pong buffer. This value of the calculation has been confirmed and verified by confirmed measurements (Fig. 8.)

The time requests of three sequential conversion tasks have been specifically measured:

1. DMA – this task contains the DMA transfer of data from the hardware FIFO memory into the internal memory (Fig. 9). This amount is necessary to form the payload for one UDP packet. ($t_{D}MA=82$ μs)
2. Framing – in this task, the actions are performed by the processor module: changes in the ping-pong buffer, generating the header of the UDP datagram, forming a UDP datagram and steps of forming an Ethernet frame, i.e., the operation of the software TCP/IP stack (Fig. 10). ($t_{framing}=24$ μs)
3. ETH – this task performs the transmission of the Ethernet frame from the internal memory of the processor module to the Ethernet peripheral interface (Fig. 11). ($t_{ETH}=148$ μs)

The total process time the process requires for completing a full cycle, consisting of three tasks: DMA transfer, forming a UDP packet, and sending the UDP packet, is:

(18) $$t_{task}=t_{DMA}+t_{framing}+t_{ETH}=82+24+148=254\;\mu \text{s}$$

Until the start of the next conversion, i.e. the next interrupt request, the processor module is in idle mode. It can perform other actions, e.g., actions based on UDP commands of the personal computer.

Based on the measured values, the reserve of the processor module can be calculated, i.e., the remaining time until the next interruption is:

(19) $$t_{reserve}=T_{rd_{UDP}}-t_{tasks}=458.3-254\approx 204\;\mu \text{s}$$

**Figure 12:**
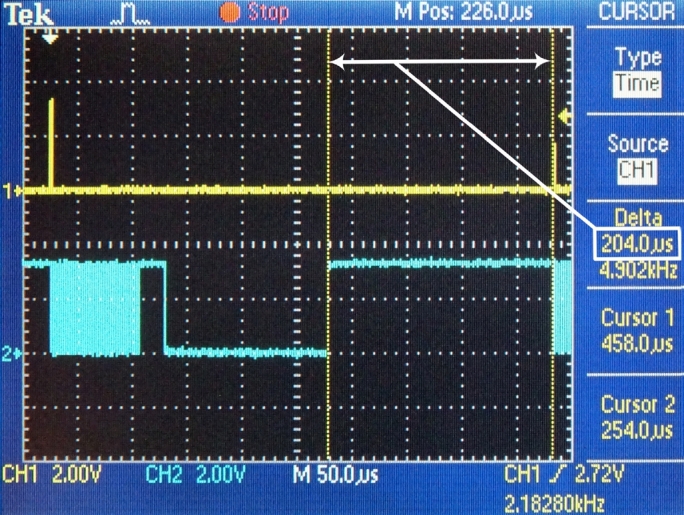
The measured value of reserve time.

**Figure 13:**
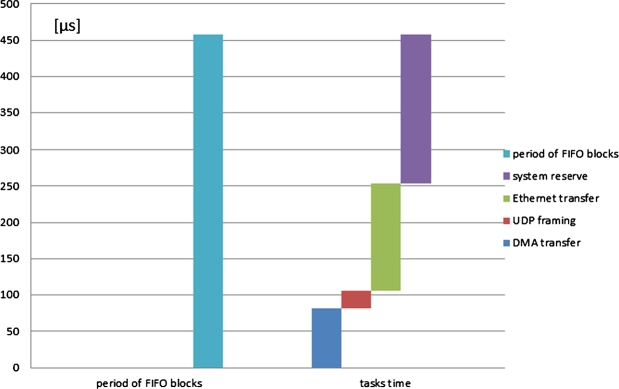
The required time of conversion tasks and reserve of one conversion.

Fig. 12 shows the measured value of the reserve time. The remaining time until the next cycle of the conversion process is approximately 200 μs (based on Eq. (19)), which, compared to 254 μs of elapsed time (Eq. (18)), totals a 43% reserve of process time. The measured and calculated values confirm the scaled processor-controlling unit is capable of converting data from the $I^{2}S$ bus in real time in the TCP/IP protocol, the required time is shown in Fig. 13.

**Figure 14:**
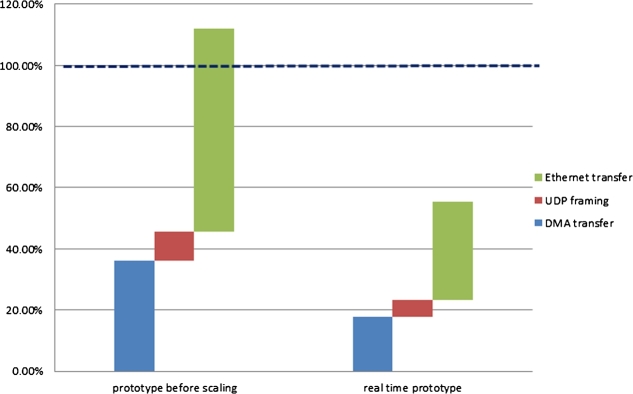
Comparing the time requirements of prototypes for one cycle of conversion.

Fig. 14 shows a comparison of the time requirements of the individual prototypes without and with scaling. The required time is depicted in relative terms, as in the form of a percentage (%).

**Figure 15:**
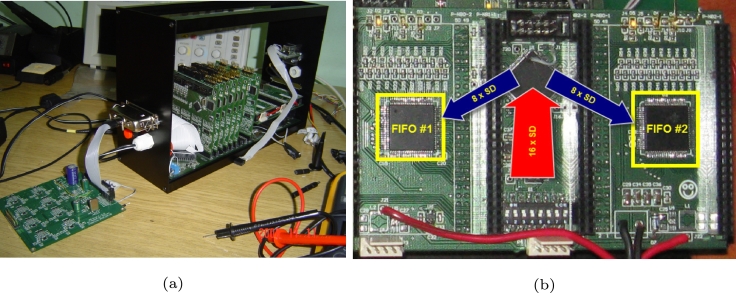
a) Stationary unit of the implemented system with the test generator; b) PCB of the protocol converter.

The left part of Fig. 14 represents the required normalized time in the case of conversion without scaling for one cycle of data conversion. In this case, the processes require more than 100% of the time, which is more than what is at its disposal. The right part of Fig. 14 shows the normalized time required by the processes if the scaled solution is used. In case of the scaled solution, less than 60% of the time is required of the total time at disposal – this solution is ideally suitable in support of the operation of the protocol converter in real time. Fig. 15.a) shows the implemented stationary unit of the system with the radio receiver, de-modulator, de-multiplexer, multichannel DSP post-process MIMD sub-system and the protocol converter. Fig. 15.b highlights a close-up of the PCB of the protocol converter.

## 4 Conclusions

The implemented model of the protocol converter was proven to perform correctly, successfully, and without loss of the conversion of data from the measuring system from the $I^{2}S$ format to UDP format which was archived on a personal computer. Data loss occurred only to the extent in which it was inherent among the UDP protocol in dedicated computer networks. The significance of loss was minimized through the log of lost UDP packets.

Using the reserve time slot according to Eq. (19), the sampling frequency can be increased up to 40 kHz in our system via modifying the embedded program parameters. As a result, the system can be employed in the 8−40kHz sampling frequency range. Similarly, the sample bit resolution can also be varied effectively. Based on the flexibility of the program parameters, the resolution can be selected in the 16−32 bit/sample range (i.e., 16, 20, 24 and 32 bit/sample resolutions can be selected, standard resolutions of $I^{2}S$).

Considering the given parameters of the measuring system, the load of the processor boards was measured at 57%. The existing reserve of 43% ensures the currently implemented processes of the protocol converter perform their tasks in real time, and without loss during transmission. Laboratory measurements and tests have proven, the implemented protocol converter fully meets all previously set requirements.

Measurements were performed to test the protocol converter. During testing, the system was fed with $I^{2}S$ signals [21] from a multichannel test generator. During testing, the level of archived data packets with an error generated by the protocol converter was lower than $10^{-6}$. These errors are interpreted to have occurred due to the use of a non-real-time operating system on the personal computer.

During the development of the hardware and software, the guiding principle was to anticipate the scaling of the system. The system has been designed in a way in which processor modules can be added to it if data from even more channels are to be accepted or a higher sampling rate is required.

The proposed structure can be used for the implementation in support of other systems in which a protocol converter needs to be built from a synchronous serial protocol into a packet protocol. The scalable structure also provides a solution for cases where a stand-alone processor does not have sufficient resources for transmitting data streams.

One possible area of further action is the application of method we have developed in the brain machine interface technique. The most important criterion for this is the introduction and application of wireless methods by Radio frequency identification (RFID) and Delay Tolerant Network (DTN) technology. Combining the method we have developed, the RFID and DTN processes allows us to create an even more complex brain machine interface [30], [31], [32].

## Declarations

### Author contribution statement

Zoltan Vizvari, Attila Toth & Bojan Kuljic: Performed the experiments; Wrote the paper.

Sari Zoltan & Mihaly Klincsik: Analyzed and interpreted the data.

Tibor Szakall: Contributed reagents, materials, analysis tools or data; Wrote the paper.

Akos Odry: Performed the experiments; Contributed reagents, materials, analysis tools or data.

Kalman Mathe: Contributed reagents, materials, analysis tools or data.

Imre Szabo & Karadi Zoltan: Conceived and designed the experiments.

Peter Odry: Conceived and designed the experiments; Contributed reagents, materials, analysis tools or data.

### Funding statement

This research has been supported and funded by projects EFOP-3.6.1-16-2016-00003, GINOP-2.3.2-15-2016-00022 and TUDFO/51757-1/2019-ITM.

### Competing interest statement

The authors declare no conflict of interest.

### Additional information

No additional information is available for this paper.
